# Antihypertensive Peptides from Milk Proteins

**DOI:** 10.3390/ph3010251

**Published:** 2010-01-19

**Authors:** Pauliina Jäkälä, Heikki Vapaatalo

**Affiliations:** Institute of Biomedicine, Pharmacology, University of Helsinki, P.O. Box 63, 00014, University of Helsinki, Finland

**Keywords:** hypertension, casein, bioactive peptides, lactotripeptides, Ile-Pro-Pro

## Abstract

Dietary proteins possess a wide range of nutritional and functional properties. They are used as a source of energy and amino acids, which are needed for growth and development. Many dietary proteins, especially milk proteins, contain physiologically active peptides encrypted in the protein sequence. These peptides may be released during gastrointestinal digestion or food processing and once liberated, cause different physiological functions. Milk-derived bioactive peptides are shown to have antihypertensive, antimicrobial, immunomodulatory, antioxidative and mineral-binding properties. During the fermentation of milk with certain lactobacilli, two interesting tripeptides Ile-Pro-Pro and Val-Pro-Pro are released from casein to the final product. These lactotripeptides have attenuated the development of hypertension in several animal models and lowered blood pressure in clinical studies. They inhibit ACE *in vitro* at micromolar concentrations, protect endothelial function *in vitro* and reduce arterial stiffness in humans. Thus, milk as a traditional food product can after certain processing serve as a functional food and carry specific health-promoting effects, providing an option to control blood pressure.

## 1. Introduction

Hypertension affects up to 30% of the adult population in most countries [1]. It is a known risk factor for cardiovascular diseases, including coronary heart disease, peripheral artery disease and stroke. Because of high prevalence and severe consequences, hypertension poses an important, world-wide health challenge. Any reduction in blood pressure, however small, is meaningful; systolic blood pressure (SBP) reduction of 10–12 mmHg and diastolic blood pressure (DBP) reduction of 5 mmHg may reduce the risk of stroke by 40%, coronary heart disease by 16% and all-cause mortality by 13% [2].

Besides pharmacological therapy, nutritional factors play a significant role in the prevention and treatment of hypertension. Dietary efforts to decrease saturated fat and sodium and increase potassium, calcium and soluble fiber intake affect positively blood pressure [3]. Although drug therapy is needed for most hypertensive patients, changes in diet and lifestyle (physical activity, weight reduction, smoking cessation) may be enough for some people with mild hypertension to decrease the blood pressure to the desired level. To enhance preventive and early treatment, a new classification of hypertension was established in 2003. Individuals with a blood pressure between normal levels and established hypertension (SBP 120–139 mmHg and DBP 80–89 mmHg) are now categorized as having ‘prehypertension’ [4].

Prevention of diseases may in the future be just as important as treatment of diseases. Interest on the possible health-promoting properties of food is increasing and more and more research is targeted at the search for new biologically active compounds in different food products. Milk and dairy products have traditionally been an important part of human nutrition. Milk is a good source of protein and essential amino acids, minerals and vitamins (especially calcium, magnesium, zinc, vitamin A and vitamin B_12_) [5]. However, fatty acid profile of non-modifiable milk is not favourable; approximately 75% of total fatty acids in milk are saturated [6].

Milk protein is cleaved to small peptide fragments during dairy processing or gastrointestinal digestion. These peptides have been shown to possess different physiological activities, such as antihypertensive, antimicrobial and immunomodulatory effects. The most studied bioactive peptides at present are those inhibiting angiotensin-converting enzyme (ACE). ACE, as a part of the renin-angiotensin system (RAS), has an important role in the regulation of blood pressure by converting angiotensin I to a potent vasoconstrictor, angiotensin II, which induces the release of aldosterone and therefore increases the sodium concentration and blood pressure further. By inhibiting ACE or by other, still poorly known mechanisms, milk-derived peptides have been shown to lower blood pressure in animal and clinical studies. In this review we give an overview of the milk-derived antihypertensive peptides, and based on animal and clinical studies, critically evaluate their usefulness in the prevention and treatment of hypertension.

## 2. Milk and Blood Pressure

Epidemiological studies have suggested that consumption of dairy products is inversely related to the risk of hypertension. The first National Health and Nutrition Examination Survey (NHANES I) showed that low consumption of milk products was related to high blood pressure [7]. Intake of dairy products, particularly low-fat products, has consistently been associated with lower blood pressure levels and reduced risk of hypertension also in other observational studies. A nine years’ follow-up study of 6,912 white, nonhypertensive men and women showed that subjects consuming three or more servings of low-fat milk per day had lower increase of blood pressure compared to those consuming less than one serving per week [8]. Also a prospective cohort study, in which 28,886 middle-aged and older (≥45 years) US women without baseline hypertension were followed for 10 years, found that intake of low-fat dairy products was inversely associated with risk of hypertension [9]. In addition, a longitudinal 12-month analysis of PREDIMED trial participants (men and women aged 55–80 and 60–80 years, respectively, at high cardiovascular risk) and their diet showed the inverse association of low-fat dairy products and blood pressure [10]. Interestingly, a prospective study of 3,157 young adults (18–30 years) followed for 10 years found that the incidence of elevated blood pressure was inversely associated with total dairy food intake, if subjects had body mass index (BMI) ≥ 25 but not in normal weight subjects [11]. 

The association of dairy consumption and blood pressure has been shown also in intervention studies. The famous Dietary Approaches to Stop Hypertension (DASH) trial with 459 normotensive or mildly hypertensive subjects showed that a diet rich in fruits, vegetables and low-fat dairy products lowers blood pressure significantly [12]. This combination diet (DASH diet) decreased SBP and DBP by 5.5 and 3.0 mmHg, respectively, more than the control diet whereas a diet containing fruits and vegetables alone produced blood pressure reductions of roughly half of this. However, other dietary alterations (e.g., reduced saturated fat) were also incorporated into the DASH diet, so the greater reduction in SBP cannot be ascribed to the milk products only. Nonetheless, other intervention studies have as well demonstrated a relationship between the intake of milk products and reduction in blood pressure [13,14,15]. 

### 2.1. Minerals

Milk is rich in potassium, magnesium and calcium [5]. A meta-analysis of 33 randomized controlled clinical trials (total 2,609 subjects) showed that increased intake of potassium reduce SBP and DBP by 3.1 mmHg and 2.0 mmHg, respectively [16]. The blood pressure lowering effects of potassium has been shown to be more marked in hypertensive subjects [17]. There is also a considerable number of studies demonstrating the effect of calcium on blood pressure. According to a meta-analysis of 40 studies (2,492 subjects), calcium supplementation (mean 1,200 mg/day) decreased SBP by 1.9 mmHg and DBP by 1.0 mmHg [18]. Thus, electrolyte content may partly explain the inverse association of consumption of milk products and blood pressure. Milk is low in sodium and consequently favorable as concerns the need for the decrease of salt intake in controlling high blood pressure.

### 2.2. Protein

Most observational studies suggest that increased intake of protein is associated with lower blood pressure and attenuated blood pressure increase over time [19,20]. Interestingly, some studies show that the beneficial effect on blood pressure results from increased intake of protein from plant rather than animal sources [21,22]. Although the number of randomized, controlled clinical trials large enough is limited, mainly positive effects of protein intake on blood pressure have been reported [23,24,25].

Bovine milk contains about 32 g/l protein of which 80 % are caseins and 20 % whey proteins [26]. Caseins can be divided into *α-*,* β-* and *κ-*casein. The whey fraction contains α-lactalbumin, β-lactoglobulin and various other proteins (e.g., immunoglobulins, lactoferrin) in smaller quantities. Antihypertensive peptide sequences have been detected both from casein and whey fractions (see below).

## 3. Generation of Antihypertensive Peptides from Milk Protein

Although casein and whey have been shown to decrease blood pressure also as such [27], research has been focused on their degradation products, peptides. Peptides may be deliberated from their parent protein by enzymatic hydrolysis during gastrointestinal digestion, fermentation of milk with proteolytic starter cultures or hydrolysis by enzymes obtained from microorganisms [28]. If the structure of the peptide is known, it is also possible to synthesize peptides by chemical synthesis, recombinant DNA technology or enzymatic synthesis [29].

### 3.1. Gastrointestinal Digestion

Physiologically active peptides are produced from several milk proteins during gastrointestinal digestion [30,31,32]. Hydrolysis may occur in various stages after ingestion of the protein. In the gastrointestinal tract, ingested proteins are hydrolysed by proteinases such as pepsin, trypsin and chymotrypsin to produce peptides of various lengths. Some proteins may be resistant to proteinases and remain intact. Thereafter, some of the peptides are further digested at the surface of epithelial cells by brush-border peptidases to produce short peptides or amino acids. Peptides may express a variety of functions either at the gastrointestinal tract, at the intestinal epithelium or after systemic absorption into circulation (for a more detailed review, see [33]).

### 3.2. Fermentation of Milk with Proteolytic Starter Cultures

In the fermentation process of milk, bioactive peptides can be generated by dairy starter cultures, which show high proteolytic activity [34]. Lactic acid bacteria utilize milk protein, especially casein, as a source of amino acids for growth [35]. For example, *Lactobacillus helveticus* and *Lactococcus lactis* strains have been shown to produce bioactive peptides [36,37,38].

### 3.3. Enzymatic Hydrolysis

Gastrointestinal enzymes, such as pepsin and trypsin may be utilized to generate bioactive peptides from whole proteins [39]. Also enzyme combinations (e.g., Alcalase™) can be used [40]. As concerns live microorganisms, proteolytic enzymes of lactic acid bacteria may be isolated, purificated and used to produce bioactive peptides from casein of different species [41]. In functional food production, use of commercially available microbial-derived proteinases and ultrafiltration membrane reactors is cost-effective and increases product yields [42].

## 4. Occurrence of Antihypertensive Peptides in Dairy Products

Antihypertensive peptides have been found in processed dairy products (cheese, milk) without any intentional functional role [43]. Lactotripeptides isoleucine-proline-proline (Ile-Pro-Pro) and valine-proline-proline (Val-Pro-Pro) have been found from sour milk [37]. Also several cheeses from Swiss origin contain the same tripeptides [44]. The concentration of Ile-Pro-Pro and Val-Pro-Pro seems to increase in the course of ripening process, reaching 100 mg/kg after 4–7 months. Whey fraction of a yoghurt-like product was found to contain a dipeptide Tyr-Pro, which produced a significant antihypertensive effect in spontaneously hypertensive rats (SHR) [45].

## 5. Animal studies

### 5.1. Casein-Derived Peptides

The antihypertensive effect of milk casein-derived peptides was first demonstrated by casein hydrolysate formed by purified proteinase from *L. helveticus* CP790 and milk fermented with the same bacteria [46]. Acute blood pressure lowering effect after oral administration was observed in SHR but not in normotensive Wistar-Kyoto (WKY) rats. The authors concluded that the peptides deliberated from casein by extracellular proteinase were responsible for the antihypertensive activity. Thereafter, the same group showed that angiotensin-converting enzyme (ACE)-inhibitory substances were produced during fermentation of milk with *L. helveticus* and *Saccharomyces cerevisiae* [36]. After isolation, these ACE-inhibitory substances were identified to be Ile-Pro-Pro-and Val-Pro-Pro. The IC_50_-values were 5 and 9 µM, respectively. These amino acid sequences are found in the primary structure of bovine β-casein (74–76 Ile-Pro-Pro and 84–86 Val-Pro-Pro) and κ-casein (108–110 Ile-Pro-Pro). Oral administration of the previously described fermented milk or pure tripeptides were shown to produce strong antihypertensive effect in SHR after a single-dose [37]. It has been found later that IC_50_-values of Ile-Pro-Pro and Val-Pro-Pro can be even lower depending on the substrate concentration used in the *in vitro* experiments [47]. Also a third tripeptide, leucine-proline-proline (Leu-Pro-Pro) found from bovine β-casein (151–153), has been shown to inhibit ACE.

Thereafter, several animal studies have been conducted by us and others to further characterize also the long-term effects of lactotripeptides Ile-Pro-Pro and Val-Pro-Pro or fermented milk products containing them. In these studies, mainly SHR [48,49,50,51,52,53] but also salt-loaded type 2 diabetic Goto-Kakizaki (GK) rats [54] and double transgenic rats (dTGR) with malignant hypertension [55] have been used.

Nakamura *et al.* [49] showed that long-term feeding with diet containing 2.5% lyophilized sour milk inhibited the development of hypertension in SHR. Rats that had received sour milk in their diet for 16 weeks had 19 mmHg lower SBP at the end of the study compared to the control group. Lower SBP was found even after 48 h replacement of the sour milk diet by the control diet, suggesting also a long-lasting antihypertensive effect of the sour milk. In this study, ACE activity in aorta was found to be decreased significantly in the sour milk group.

Further evidence from the ACE-inhibition was gained from the study of Masuda *et al.* [56]. After receiving a single-dose of Calpis™ sour milk, ACE activity was decreased in SHR aorta significantly. Furthermore, tripeptides Ile-Pro-Pro and Val-Pro-Pro were detected by HPLC in the solubilized fraction from the abdominal aorta. Interestingly, tripeptides were detected from aortas of SHR but not WKY given the sour milk. As ACE activity was significantly higher in aortas of SHR compared to WKY, authors suggest that there may have not been sufficient amounts of ACE in WKY aorta to capture tripeptides so that they would retain in the aortic tissue to be detected in the HPLC analysis.

After the first findings concerning antihypertensive effects of lactotripeptides Ile-Pro-Pro and Val-Pro-Pro, Sipola *et al*. [50,51], Jauhiainen *et al*. [52,55] and Jäkälä *et al*. [53,54] have studied the long-term effects of these tripeptides and fermented milk products containing them in more detail. The development of hypertension has attenuated significantly in rats receiving Evolus^®^, a fermented milk product containing Ile-Pro-Pro and Val-Pro-Pro. The attenuation of systolic blood pressure has been 12–21 mmHg in SHR, 10 mmHg in high-salt fed GK rats and 19 mmHg in dTGR in comparison to control group. Pure tripeptides have not produced as strong antihypertensive effect as the milk products containing them [50,52]. Minerals (calcium, potassium) present in the milk product have likely contributed to the more pronounced antihypertensive effect. However, in the study of Jauhiainen *et al.* [52] minerals alone did not attenuate the development of blood pressure as much as the fermented milk product. The bioavailability of peptides may be better from milk in comparison to water and improved by other milk components. 

Also a long-term treatment with fermented milk products containing Ile-Pro-Pro and Val-Pro-Pro has shown to have effects on renin-angiotensin-aldosterone-system. In SHR, plasma renin activity increased after treatment with tripeptides [51]. In addition, treatment with a fermented milk product containing both lactotripeptides and plant sterols decreased serum ACE activity [53]. Furthermore, in salt-loaded GK rats, lactotripeptide-containing fermented milk products decreased serum ACE and aldosterone levels [54].

It seems that the antihypertensive effect of lactotripeptides is dose-related, as shown in the study of Sipola *et al*. [51], where two fermented milk products containing different amounts of lactotripeptides were studied. Also if the treatment either with pure tripeptides or fermented milk product has been terminated, the blood pressure of treated rats has gradually increased to the same level as with the control rats [50]. 

Effect of long-term intake of lactotripeptides on vascular function has been assessed in several studies after the blood pressure monitoring has been completed [52,53,54]. In the study of Jauhiainen *et al*. [52], mesenteric arteries and aortas of rats that had received minerals and lactotripeptides showed improved endothelium-dependent relaxation. In the study with salt-loaded Goto-Kakizaki rats [54], endothelial function of mesenteric arteries was strongly impaired in all groups, but significantly improved endothelium-dependent relaxations were still observed after treatment with different fermented milk products. Also an *in vitro* study with isolated SHR mesenteric arteries showed protection of endothelial function after incubation with tripeptides Ile-ProPro and Val-Pro-Pro for 24 h [57].

Besides the most extensively studied lactotripeptides, also other antihypertensive peptides have been found from casein. Peptides obtained by enzymatic hydrolysis of casein with pepsin and corresponding to α_s1_-casein f(90–94) (RYLGY), α_s1_-casein f(143–149) (AYFYPEL) and α_s2_-casein f(89–95) (YQKFPQY) exerted antihypertensive activity after oral administration to SHR [58]. They also showed to inhibit ACE by IC_50_ values of 0.7, 6.6 and 20.1 µM, respectively. In addition, ß-casein f(133–138) peptide (LHLPLP), identified from milk fermented with *Enterococcus faecalis*, showed a significant antihypertensive effect in SHR [59].

### 5.2. Whey-Derived Peptides

In addition to casein-derived peptides, antihypertensive peptides have been found also from the whey fraction of milk protein. α-Lactorphin (Tyr-Gly-Leu-Phe) and β-lactorphin (Tyr-Leu-Leu-Phe) can be released from milk whey proteins α-lactalbumin and β-lactoglobulin, respectively, in enzymatic proteolysis by gastric and pancreatic enzymes [60]. Nurminen *et al*. [61] observed that α-lactorphin produced a transient, dose-dependent blood pressure lowering effect, which was abolished by a specific opioid receptor antagonist, naloxone. Both tetrapeptides improved arterial function in an *in vitro* study by Sipola *et al*. [62]. Another tetrapeptide from β-lactoglobulin, β-lactosin B (Ala-Leu-Pro-Met) showed strong antihypertensive effect in SHR as well [63]. Also a proteinase K-digested whey of cheese origin was shown to decrease blood pressure in SHR after single-dose administration [64]. From the digest, the peptide showing the strongest antihypertensive activity was found to be tripeptide Ile-Pro-Ala, originating from β-lactoglobulin. General therapeutic applications of whey proteins have recently been reviewed by Marshall [65] and Saito [66]. 

## 6. Clinical Studies

### 6.1. Casein-Derived Peptides

Recently, two meta-analyses on antihypertensive peptides derived from food sources have been performed [67,68]. Pripp [67] included 15 clinical trials in the analysis, of which 13 trials concerned milk-derived peptides. Casein-derived lactotripeptides Ile-Pro-Pro and Val-Pro-Pro were studied in 9 of them. Xu *et al*. [68] had 12 trials in the analysis and the intervention in all of them contained lactotripeptides.

Significant decreases of 4.8 mmHg in SBP and 2.2 mmHg in DBP were found in the meta-analysis of Xu *et al*. [68]. When a stratified meta-analysis of trials with lactotripeptides were performed in the study of Pripp [67], the result was very similar; 4.6 and 2.2 mmHg in SBP and DBP, respectively. All the trials included in these analyses had lasted at least 4 weeks and were performed in Finland or Japan. Based on the baseline values, all the subjects in the trials could have been regarded as prehypertensive or hypertensive [4]. These two analyses provided evidence that lactotripeptides Ile-Pro-Pro and Val-Pro-Pro have antihypertensive effects in prehypertensive and mildly hypertensive subjects.

However, after these meta-analyses several further clinical trials on lactotripeptide-containing products have been published [69,70,71,72,73,74,75,76]. Besides Finland and Japan, studies have now been conducted also in the Netherlands and Scotland. Five of these newer trials report hypotensive effects by treatment with lactotripeptides; SBP decreases of 3 mmHg [72], 6 mmHg [74,75] and as large as 16 mmHg have been observed [73]. However, Dutch studies by Engberink *et al*. [69], van der Zander *et al*. [70] and van Mierlo *et al*. [76] did not find any significant effect either on SBP or DBP by treatment with lactotripeptide-containing products.

The effect of lactotripeptides on blood pressure has been studied mainly in long-term clinical trials. However, van der Zander *et al*. [71] studied the acute hypotensive effect by following blood pressure over a period of 8 hours after ingestion of a lactotripeptide-containing milk product. A significant decrease of 2 mmHg in SBP was observed. Other studies have lasted for 4 to 21 weeks. As Xu *et al*. [68] reported in their review, the hypotensive effect of lactotripeptide-containing products becomes more obvious as the intervention is lengthened. Some trials of long duration have reported blood pressure values also during the intervention. When this data was gathered by Xu *et al.* [68], no significant blood pressure lowering effect was seen after 2 weeks’ of intervention, but already after 4 weeks the SBP had decreased by 2.2 mmHg, which differed significantly from the baseline values. The blood pressure lowering effect seems to be greater in patients with higher baseline blood pressure levels. Studies involving a follow-up period show that when the treatment is terminated, blood pressure returns gradually to the baseline within 2–4 weeks [77,78,79].

In contrast to the published data from animal studies in which lactotripeptides have shown clear antihypertensive effects in different hypertension models, human data is more contradictory. Because most of the clinical studies are carried out by or at least with industry, not all data concerning e.g., the product composition or manufacturing is available. This makes the critical evaluation of the findings difficult. However, some suggestions for these controversies will be discussed in the following.

Lactotripeptides have been given in several forms in different studies. In most clinical studies, the test products have consisted of sour milk prepared by fermenting skim milk with *L. helveticus* and/or *S. cerevisiae*. As a placebo regular sour milk or artificially acidified milk has been used. In some studies, test products have consisted of powdered fermented milk incorporated into tablets [80,81] or casein hydrolysate incorporated into capsules [82]. Also fruit juice has been used as a carrier [83]. In a recent study by Turpeinen *et al*. [75], lactotripeptides were given in a spread. Corresponding placebo products have been prepared but without the active ingredients. The components of a milk product may influence the peptide absorption, act directly on blood pressure (e.g., minerals) or contain other bioactive peptides in addition to Ile-Pro-Pro and Val-Pro-Pro. Therefore the results from different trials with different products are not directly comparable, especially when the effects on a biological variable are small.

The second point to be discussed is the dose. The lowest dose that has been shown to be effective in humans is 3.07 mg of lactotripeptides/day [83]. The highest tested dose has been 52.5 mg of lactotripeptides/day [84]. When capsules have been used, the dose is easy to be determined. However, when fresh, liquid products have been used, the concentrations of the peptides may vary, especially if regular product control during the long-term trial has not been conducted. 

The third issue is blood pressure measurement; office, home or 24-hour ambulatory blood pressure registration. Even when the golden standard 24 h-measurement has been used, the analysis of the results and statistics used should be carefully evaluated also by the reader. 

The fourth issue is the production process of the peptides. In clinical trials, test products have contained lactotripeptides produced by fermentation of milk by bacteria of different species or strains (e.g., *L. helveticus* LBK-16H or CM4) or hydrolysis by different enzymes (e.g., *Aspergillus oryzae*). It is clear that these various processes do not necessarily produce active components of the same kind and amount. Attention has often been paid only to Ile-Pro-Pro and Val-Pro-Pro and their analysis from the final product.

The number of the subjects in individual trials has been quite limited even when summarized for meta-analysis (n = 623 in the meta-analysis of Xu *et al*. [68], for example). Interestingly, the antihypertensive effect of the lactotripeptide products, although in some studies with quite high peptide doses, has been evidenced especially in Japan and Finland, but not e.g., in the Netherlands. Whether this is related to the, age, lifestyle or nutritional factors of the test subjects is not known. The influence of subject background is discussed also in the review of Boelsma and Kloek [85].

Finally, many reports on clinical trials have concentrated mainly on the changes in blood pressure. Vascular effects and other variables, e.g., in clinical chemistry, have received less attention especially during the intervention. All these aspects should be taken into consideration when the message and the value of single clinical intervention study are evaluated e.g., for a meta-analysis. To get collective data on the clinical studies of antihypertensive peptides, the reader is referred to the two abovementioned meta-analyses.

### 6.2. Whey-Derived Peptides

Whey proteins and whey-derived peptides have been of less interest especially in the clinical study field. Hydrolyzed whey protein supplement decreased blood pressure of prehypertensive or stage I hypertensive subjects by 8.0 mmHg of systolic and 5.5 mmHg of diastolic blood pressure after 6 weeks of treatment [86]. In contrast, milk drink supplemented with whey powder was not found to reduce blood pressure in mildly hypertensive subjects after 12 weeks’ consumption [87]. Thus, more intervention studies are needed to confirm the possible clinical benefits of the whey-derived peptides.

## 7. Bioavailability

Bioavailability of milk-derived bioactive peptides has been questioned. Normally, peptides are rapidly metabolized to the constituent amino acids by brush border membrane peptidases after oral dosing and the absorption and bioavailability remains very low. However, there is data demonstrating that at least di- and tripeptides may absorb intact, enter the circulation and produce systemic effects [88,89]. Bioactive peptides may be absorbed via carrier-mediated transport or paracellular diffusion [33]. Apparently, short tripeptides are actively transported *via* a specific transporter (PepT1) and oligopeptides via the paracellular route. 

As regards antihypertensive peptides, Foltz *et al*. [89] investigated the transport of Ile-Pro-Pro and Val-Pro-Pro by using three different absorption models and demonstrated that these tripeptides are transported in small amounts intact across the barrier of the intestinal epithelium. The major transport mechanisms of Ile-Pro-Pro and Val-Pro-Pro were demonstrated to be paracellular transport and passive diffusion. Also a study conducted in humans showed that Ile-Pro-Pro and Leu-Pro-Pro were detectable from plasma after ingestion of tripeptide-enriched yoghurt [90]. The C_max_ of Ile-Pro-Pro rose up to almost nanomolar level and the T_max_ was reached after ~40 min. Interestingly, measurable plasma Ile-Pro-Pro concentrations were also found after ingestion of a placebo yogurt beverage without added tripeptides. This has been suggested to be due to the generation of Ile-Pro-Pro in the intestinal tract from milk proteins by luminal or brush border peptidases. In conscious pigs, Ile-Pro-Pro, Val-Pro-Pro and Leu-Pro-Pro reached the blood circulation intact after intragastric administration (4.0 mg/kg of each tripeptide) and maximal plasma concentrations were about 10 nmol/l [91]. Half-lives of absorption and elimination were only few minutes.

Many discovered ACE-inhibitory peptides have a Pro or Pro-Pro residue at the C-terminal end [36,45]. It has been reported that tripeptides containing a C-terminal proline-proline bond are usually resistant to human proteolytic enzymes (for review see [92]). Consequently, there is a strong possibility that these peptides reach the circulation and target sites intact and exert also systemic effects, as has been shown in studies reviewed above. As regards casein-derived Ile-Pro-Pro, Jauhiainen *et al*. [93] used radiolabelled tripeptide and showed that it absorbed partly intact from the gastrointestinal tract after a single oral dose to rats. Considerable amounts of radioactivity were found from several tissues, e.g., liver, kidney and aorta. The excretion of Ile-Pro-Pro was slow; even after 48 hours the radiolabelled peptide had not been completely excreted. Ile-Pro-Pro did not bind to albumin or other plasma proteins *in vitro*. Considering this and the long-lasting retention of the radioactivity in the tissues, accumulation of Ile-Pro-Pro may occur in sufficient concentrations to cause blood pressure lowering effects e.g., by ACE-inhibition in the vascular wall. 

## 8. Mechanisms

As concerns the antihypertensive mechanisms of milk-derived peptides, most evidence has been gained from ACE-inhibition. *In vitro*, several peptides of different lengths have been shown to inhibit ACE at micromolar concentrations [36,47,58,59]. *In vivo*, serum ACE levels have decreased after long-term treatment of rats with fermented milk products containing lactotripeptides Ile-Pro-Pro and Val-Pro-Pro [53,54]. Furthermore, other positive effects on renin-angiotensin system have also been reported in animal studies [51,54]. 

However, clinical studies have failed to show the ACE-inhibitory effect consistently. Thus, other mechanisms behind the observed blood pressure lowering effect may exist as well. In a few studies, the effect of lactotripeptides on arterial function has been evaluated. *In vitro*, Ile-Pro-Pro and Val-Pro-Pro protected endothelial function of isolated rat mesenteric arteries during 24 h incubation [57]. In clinical studies, different methods to evaluate endothelial function have been used. Ambulatory arterial stiffness index (AASI) can be calculated from 24-hour blood pressure recordings, and it has been shown to be an independent predictor of cardiovascular mortality [94]. Another predictor of cardiovascular outcomes is aortic augmentation index (AIx), for which pulse waveform analysis is needed [95]. In the study of Jauhiainen *et al*. [96], a significant improvement in AASI was observed after a 10-week treatment with *L. helveticus* fermented milk. In another study, AIx was decreased after 6 months’ treatment with the same product [97]. Administration of casein hydrolysate containing Ile-Pro-Pro and Val-Pro-Pro 4 times a day for 1 week in capsules increased maximum blood flow of upper forearm during reactive hyperemia [82], thus demonstrating an improvement in the vascular endothelial dysfunction in subjects with mild hypertension. Interestingly, this effect was obviously not related to a blood pressure-lowering effect, as no significant changes were detected in systemic blood pressure.

Although significant antihypertensive effects have been obtained, blood pressure lowering effects of e.g., Ile-Pro-Pro and Val-Pro-Pro seem to be quite small, if compared to ACE-inhibitory drugs. More thorough mechanistic research is probably needed to detect the small changes in the factors affecting blood pressure and vascular tone to show the exact mechanisms also *in vivo*. Yamaguchi *et al*. [98] studied effects of a 5-day repeated administration of lactotripeptides Ile-Pro-Pro and Val-Pro-Pro on gene expression of SHR abdominal aorta using DNA microarray analysis. Overall, changes in the gene expression were small, but significant increases were detected for endothelial nitric oxide synthase (eNOS) and connexin 40 (gap junction 40) genes. The expression of these two genes, important in the regulation of blood pressure, is restored in the aortic tissue of hypertensive animals after treatment with ACE-inhibitors as well [99,100].

## 9. Biochemical Aspects

Many of the antihypertensive peptides possess ACE-inhibitory activity. ACE (peptidyl-dipeptidase A, EC 3.4.15.1) is an enzyme, which cleaves the carboxy terminal His-Leu from decapeptide angiotensin I to produce octapeptide angiotensin II, a highly potent vasoconstrictor [101]. The endothelial ACE contains two homologous domains, N- and C-terminal domains, which are both catalytically active [102]. ACE-inhibitors may prefer either domain or act on both. However, the C-terminal domain seems to be necessary for blood pressure regulation and is the dominant angiotensin-converting site [103].

The majority of milk-derived ACE-inhibitory peptides have relatively low molecular mass and short chain. This is consistent with findings of Natesh *et al*. [103], which demonstrated that large peptide molecules do not fit into the active site of ACE. It seems that peptide binding to ACE is strongly influenced by the C-terminal tripeptide sequence of the peptide. Many ACE-inhibitory peptides have hydrophobic amino acid residues at each of the three C-terminal positions and proline at the C-terminal end [104]. However, *in vitro* observed ACE-inhibitory activity of a peptide is not always directly related to hypotensive effect *in vivo*, which may be due to degradation in the gastrointestinal tract. *Vice versa*, active, ACE-inhibitory fragments may be generated in the body from longer precursor peptides, which do not inhibit ACE as such.

Although ACE-inhibitory effect of a hypotensive peptide could be demonstrated *in vitro*, question on the true antihypertensive mechanism may still remain. For example, Wuerzner *et al*. [105] studied the *in vivo* ACE-inhibition of a fermented milk product containing lactotripeptides Ile-Pro-Pro and Val-Pro-Pro, which have repeatedly been shown to inhibit ACE *in vitro* and also *in vivo* in some animal studies described above. After a 7-day administration of the product, no changes were detected in plasma AcSDKP (a marker of the specific ACE-activity of the N-terminal domain), ACE activity or active renin concentrations. A small, transient increase was detected in urine AcSDKP. The authors concluded that neither plasma nor endothelial ACE was inhibited and no specific effect was seen on N-terminal or C-terminal ACE-domains. However, it should be taken into account that the study was conducted in normotensive, healthy individuals, who normally do not respond either to antihypertensive peptides or ACE-inhibitory drugs.

Limited information on the relationships between structure and activity of milk-derived antihypertensive (ACE-inhibitory) peptides is available, but a few studies have been performed to address this by quantitative structure-activity relationships (QSAR) modeling. A relationship between increased hydrophobicity of the amino acid in C-terminal position, decreased side chain size of amino acid next to the C-terminal position and ACE-inhibition of peptides up to six amino acids in length was found by Pripp *et al*. [106]. As the length of the peptide chain increased, this relationship decreased, likely because steric effects begin to have a stronger influence. According to Wu *et al*. [107], the most favorable tripeptide structure contains hydrophobic amino acid in the N-terminal, positively charged amino acid in the middle position and aromatic amino acid in the C-terminal. As concerns ACE-inhibitory dipeptides, amino acid residues with large side chains as well as hydrophobic side chains are preferred. Foltz *et al*. [108] included also peptide stability and permeability in their analysis and showed that N-terminal amino acid residues Asp, Gly and Pro as well as C-terminal residues Pro, Ser, Thr and Asp stabilized peptides towards luminal enzymatic peptide hydrolysis. Many Pro-containing dipeptides, such as Ile-Pro, Arg-Pro and Lys-Pro exhibited high ACE-inhibitory activity *in vitro* and were demonstrated to possess high intestinal stability as well.

## 10. Safety Aspects

Cow’s milk as an important part of human nutrition has a safe reputation. After ingestion, milk proteins are hydrolyzed in the gastrointestinal tract resulting in the release of peptide sequences of different lengths. Thus, human body is continuously exposed to protein hydrolysates without experiencing any adverse events. Furthermore, FDA lists protein hydrolysates as “generally recognized as safe” (GRAS) [109]. However, in the EU, no health claims or GRAS status have been attributed to milk protein hydrolysate-containing products yet [110].

Drugs acting as ACE-inhibitors, such as captopril and enalapril, possess some common adverse effects, which could theoretically concern also milk-derived antihypertensive peptides because of their ACE-inhibitory activity. ACE-inhibitors modulate renin-angiotensin system and cough, hypotension and hyperkalemia are the most frequently reported adverse effects [111]. However, dozens of clinical studies with different kinds of antihypertensive peptide products have been performed and no treatment-related safety concerns have appeared [68,80,84]. ACE-inhibitors are not recommended during pregnancy, because it has been suggested that they can produce fetopathy characterized by fetal hypotension, disruption in the development of fetal kidney and reduction in the production of amniotic fluid [112,113]. In toxicological studies in animals, neither casein hydrolysate nor Val-Pro-Pro showed any specific target organ toxicity [114]. There was no evidence to support establishment of either the Lowest Observed Effect Level (LOEL) or Maximally Tolerated Dose (MTD); both being greater than 2 g/kg/day. Similarly, no adverse effects related to casein-derived tripeptides were seen in a subchronic (90-day) repeated-dose toxicity study with rats or in a pre-natal development study with rabbits [115]. In this study the tripeptide product was obtained by hydrolysis of milk casein with an enzyme preparation derived from *A. oryzae.* Also the study by Ponstein-Simarro Doorten *et al*. [116] showed that an Ile-Pro-Pro-containing casein hydrolysate was neither mutagenic nor clastogenic *in vitro* and did not produce any adverse effects in a 90-day repeated-dose oral toxicity study in Wistar rats up to 141-fold higher doses than the anticipated intake as a functional food ingredient.

## 11. Milk Products as Functional Foods

As the interest on foods possessing health-promoting or disease-preventing properties has been increasing, more emphasis must have been put on the legal regulation of the health claims attached to the products. Authorities around the world have had to develop systematic approaches for review and assessment of scientific data. Evidence on the beneficial effects of a functional food product should be enough detailed, extensive and conclusive for the use of a health claim in the product labeling and marketing. Besides being based on generally accepted scientific evidence, the claims should be well understood by the average consumer.

Health claim legislation varies between the countries and is still in transient state. In the EU, the new European Regulation on nutrition and health claims came into force on January 2007 [117]. Reduction of disease risk claims and claims referring to children’s development and health are addressed in Article 14 as other health claims belong to Article 13. European Food Safety Authority (EFSA), which provides scientific advice to the European Commission (EC), has taken thereafter a strict attitude towards the scientific data adequate for the use of a health claim. After receiving a draft list from EC with 4,185 claims to be evaluated, EFSA started to review the evidence and to ask for clarifications from the product owners, if needed. The first series of opinions on health claims was delivered October 2009 including opinions on 523 health claims. For approximately one third of the claims the outcomes of the evaluations were favorable. Final deadline for all opinions was set for January 2010, but will probably be delayed.

One of the main objectives of the new regulation has been to ensure that consumers are not misled and that health claims actually promote healthier choices. This means that a product using a health claim has also otherwise favorable nutrient profile [117]. Health claims should not mask the overall nutritional status of a food product.

After the new legislation came into force, EFSA has been criticized, especially by the industry, for being too strict and hindering innovations. Authorities seem to take into account mainly clinical studies, when evaluating the applications. The problem in the functional food research is that the differences in human studies are usually quite small between the active and placebo treatment, so the study population should be enormously large to prove the efficacy firmly. We suggest that more emphasis should be therefore given to preclinical studies, providing that results are consistent and that effects are shown repeatedly and by several research groups. With sound hypotheses and well-defined methods these studies can give valuable insight into the mechanisms of action as well.

For the meantime, antihypertensive lactotripeptide-containing milk products do not have approved health claims in the EU. EFSA considered the evidence on the antihypertensive effect of lactotripeptides to be insufficient. Although a number of clinical studies showing positive results have been published, there are a few studies showing no effect on blood pressure. However, claims such as “helps to control blood pressure” or “helps to keep blood pressure at healthy levels” could perhaps be attached to the lactotripeptide-containing products in the future, if more supporting data is gained and a positive opinion from EFSA is reached.

## 12. Conclusions

This review concentrates on critical evaluation of the present published data on the background, experimental and clinical data on milk protein-derived antihypertensive peptides. The blood pressure lowering effect has been confirmed in different animal models of human hypertension. There is some evidence for their beneficial effect on vasculature as well. All these effects could have been related to ACE-inhibition.

Clinical evidence for the antihypertensive effect of the lactotripeptides is more controversial. The minor reduction of blood pressure in a small number of heterogenous subjects with varying degree of hypertension with different doses and products may explain the contradictory observations. Despite of all the limitations of the present data, we suggest that products containing lactotripeptides offer a valuable option as a non-pharmacological, nutritional treatment of elevated blood pressure.

## References

[B1] Kearney P.M., Whelton M., Reynolds K., Muntner P., Whelton P.K., He J. (2005). Global burden of hypertension. Lancet.

[B2] Collins R., MacMahon S. (1994). Blood pressure, antihypertensive drug treatment and the risks of stroke and coronary heart disease. Br. Med. Bull..

[B3] Krousel-Wood M.A., Muntner P., He J., Whelton P.K. (2004). Primary prevention of essential hypertension. Med. Clin. North. Am..

[B4] Chobanian A.V., Bakris G.L., Black H.R., Cushman W.C., Green L.A., Izzo J.L., Jones D.W., Materson B.J., Oparil S. (2003). National Heart, Lung, and Blood Institute Joint National Committee on Prevention, Detection, Evaluation, and Treatment of High Blood Pressure; National High Blood Pressure Education Program Coordinating Committee. The seventh report of the Joint National Committee on Prevention, Detection, Evaluation, and Treatment of High Blood Pressure: the JNC 7 report. JAMA.

[B5] USDA National Nutrient database for Standard Reference.

[B6] Mansbridge R.L., Blake J.S. (1997). Nutritional factors affecting the fatty acid composition of bovine milk. Br. J. Nutr..

[B7] McCarron D.A., Morris C.D., Henry H.J., Stanton J.L. (1984). Blood pressure and nutrient intake in the United States. Science.

[B8] Alonso A., Steffen L.M., Folsom A.R. (2009). Dairy intake and changes in blood pressure over 9 years: The ARIC study. Eur. J. Clin. Nutr..

[B9] Wang L. (2008). Dietary intake of dairy products, calcium, and vitamin D and the risk of hypertension in middle-aged and older women. Hypertension.

[B10] Toledo E., Delgado-Rodríguez M., Estruch R., Salas-Salvadó J., Corella D., Gomez-Gracia E., Fiol M., Lamuela-Raventós R.M., Schröder H., Arós F., Ros E., Ruíz-Gutiérrez V., Lapetra J., Conde-Herrera M., Sáez G., Vinyoles E., Martínez-González MA. (2009). Low-fat dairy products and blood pressure: Follow-up of 2290 older persons at high cardiovascular risk participating the PREDIMED study. Br. J. Nutr..

[B11] Pereira M.A., Jacobs D.R., Van Horn L., Slattery M.L., Kartashov A.I., Ludwig D.S. (2002). Dairy consumption, obesity, and the insulin resistance syndrome in young adults: The CARDIA study. JAMA.

[B12] Appel L.J., Moore T.J., Obarzanek E., Vollmer W.M., Svetkey L.P., Sacks F.M., Bray G.A., Vogt T.M., Cutler J.A., Windhauser M.M., Lin P.H., Karanja N. (1997). A clinical trial of the effects of dietary patterns on blood pressure. DASH Collaborative Research Group. N. Engl. J. Med..

[B13] van Beresteijn E.C., van Schaik M., Schaafsma G. (1990). Milk: Does it affect blood pressure? A controlled intervention study. J. Intern. Med..

[B14] Buonopane G.J., Kilara A., Smith J.S., McCarthy R.D. (1992). Effect of skim milk supplementation on blood cholesterol concentration, blood pressure, and triglycerides in a free-living human population. J. Am. Coll. Nutr..

[B15] Hilary Green J., Richards J.K., Bunning R.L. (2000). Blood pressure responses to high-calcium skim milk and potassium-enriched high-calcium skim milk. J. Hypertens..

[B16] Whelton P.K., He J., Cutler J.A. (1997). Effects of oral potassium on blood pressure. Meta-analysis of randomized controlled clinical trials. JAMA.

[B17] Geleijnse J.M., Kok F.J., Grobbee D.E. (2003). Blood pressure response to changes in sodium and potassium intake: A metaregression analysis of randomized trials. J. Hum. Hypertens..

[B18] van Mierlo L.A., Arends L.R., Streppel M.T., Zeegers M.P., Kok F.J., Grobbee D.E., Geleijnse J.M. (2006). Blood pressure response to calcium supplementation: A meta-analysis of randomized controlled trials. J. Hum. Hypertens..

[B19] Obarzanek E., Velletri P.A., Cutler J.A. (1996). Dietary protein and blood pressure. JAMA.

[B20] He J., Whelton P.K. (1999). Effect of dietary fiber and protein intake on blood pressure: A review of epidemiological evidence. Clin. Exp. Hypertens..

[B21] Elliot P., Stamler J., Dyer A.R., Appel L., Dennis B., Kesteloot H., Ueshima H., Okayama A., Chan Q., Garside D.B., Zhou B. (2006). Association between protein intake and blood pressure. The INTERMAP Study. Arch. Intern. Med..

[B22] Wang Y.F., Yancy W.S., Yu D., Champagne C., Appel L.J., Lin P.H. (2008). The relationship between dietary protein intake and blood pressure: Results from the PREMIER study. J. Hum. Hypertens..

[B23] Burke V., Hodgson J.M., Beilin L.J., Giangiulioi N., Rogers P., Puddey I.B. (2001). Dietary protein and soluble fiber reduce ambulatory blood pressure in treated hypertensives. Hypertension.

[B24] Appel L.J., Carey V.J., Obarzanek E., Swain J.F., Miller E.R., Conlin P.R., Erlinger T.P., Rosner B.A., Laranjo N.M., Charleston J., McCarron P., Bishop L.M., OmniHeart Collaborative Research Group. (2005). Effects of protein, monounsaturated fat, and carbohydrate intake on blood pressure and serum lipids: Results of the OmniHeart randomized trial. JAMA.

[B25] He J., Gu D., Wu X., Chen J., Duan X., Chen J., Whelton P.K. (2005). Effect of soybean protein on blood pressure: A randomized, controlled trial. Ann. Intern. Med..

[B26] Haug A., Hostmark A.T., Harstad O.M. (2007). Bovine milk in human nutrition – a review. Lipids Health. Dis..

[B27] Pal S., Ellis V. (2009). The chronic effects of whey proteins on blood pressure, vascular function, and inflammatory markers in overweight individuals. Obesity.

[B28] Phelan M., Aisling A., FitzGerald R.J., O’Brien N.M. (2009). Casein-derived bioactive peptides: Biological effects, industrial uses, safety aspects and regulatory status. Int. Dairy J..

[B29] Gill I., López-Fandiño R., Jorba X., Vulfson E.N. (1996). Biologically active peptides and enzymatic approaches to their production. Enzyme. Microb. Technol..

[B30] Chabance B., Marteau P., Rambaud J.C., Migliore-Samour D., Boynard M., Perrotin P., Guillet R., Jollès P., Fiat A.M. (1998). Casein peptide release and passage to the blood in humans during digestion of milk and yoghurt. Biochimie.

[B31] Parrot S., Degraeve P., Curia C., Martial-Gros A. (2003). In vitro study on digestion of peptides in Emmental cheese: Analytical evaluation and influence on angiotensin I converting enzyme inhibitory peptides. Nahrung.

[B32] Hernández-Ledesma B., Amigo L., Ramos M., Recio I. (2004). Angiotensin converting enzyme inhibitory activity in commercial fermented products. Formation of peptides under simulated gastrointestinal digestion. J. Agric. Food Chem..

[B33] Shimizu M. (2004). Food-derived peptides and intestinal functions. BioFactors.

[B34] Christensen J.E., Dudley E.G., Pederson J.A., Steele J.L. (1999). Peptidases and amino acid catabolism in lactic acid bacteria. Antonie van Leeuwenhoek.

[B35] Juillard V., Guillot A., Le Bars D., Gripon J.C. (1998). Specificity of milk peptide utilization by *Lactococcus lactis*. Appl. Environ. Microbiol..

[B36] Nakamura Y., Yamamoto N., Sakai K., Okubo A., Yamazaki S.: Takano (1995). Purification and characterization of angiotensin I-converting enzyme inhibitors from sour milk. J. Dairy Sci..

[B37] Nakamura Y., Yamamoto N., Sakai K., Takano T. (1995). Antihypertensive effect of sour milk and peptides isolated from it that are inhibitors to angiotensin I-converting enzyme. J. Dairy Sci..

[B38] FitzGerald R.J., Murray B.A. (2006). Bioactive peptides and lactic fermentations. Int. J. Dairy Technol..

[B39] Pihlanto-Leppälä A., Koskinen P., Piilola K., Tupasela T., Korhonen H. (2000). Angiotensin I-converting enzyme inhibitory properties of whey protein digests: Concentration and characterization of active peptides. J. Dairy Res..

[B40] Wu J., Ding X. (2002). Characterization of inhibition and stability of soy-protein derived angiotensin I-converting enzyme inhibitory peptides. Food Res. Int..

[B41] Minervini F., Algaron F., Rizzello C.G., Fox P.F., Monnet V., Gobbetti M. (2003). Angiotensin I-converting-enzyme-inhibitory and antibacterial peptides from *Lactobacillus helveticus* PR4 proteinase-hydrolyzed caseins of milk from six species. Appl. Environ. Microbiol..

[B42] Korhonen H., Pihlanto A. (2003). Food-derived bioactive peptides – Opportunities for designing future foods. Curr. Pharm. Des..

[B43] Korhonen H.J. (2009). Milk-derived bioactive peptides: From science to applications. J. Functional Foods.

[B44] Meyer J., Bütikofer U., Walther B., Wechsler D., Sieber R. (2009). Hot topic: Changes in angiotensin-converting enzyme inhibition and concentrations of the tripeptides Val-Pro-Pro and Ile-Pro-Pro during ripening of different Swiss cheese varieties. J. Dairy Sci..

[B45] Yamamoto N., Maeno M. (1999). Purification and characterization of an antihypertensive peptide from a yogurt-like product fermented by *Lactobacillus helveticus* CPN4. J. Dairy Sci..

[B46] Yamamoto N., Akino A., Takano T. (1994). Antihypertensive effect of the peptides derived from casein by an extracellular proteinase from *Lactobacillus helveticus* CP790. J. Dairy Sci..

[B47] Lehtinen R., Jauhiainen T., Kankuri E., Lindstedt K., Kovanen P.T., Kerojoki O., Korpela R., Vapaatalo H. (2010). Effects of milk casein-derived tripeptides Ile-Pro-Pro, Val-Pro-Pro, and Leu-Pro-Pro on enzymes processing vasoactive precursors *in vitro*. Arzneim. Forsch/Drug Res..

[B48] Yamamoto N., Akino A., Takano T. (1994). Antihypertensive effects of different kinds of fermented milk in spontaneously hypertensive rats. Biosci. Biotech. Biochem..

[B49] Nakamura Y., Masuda O., Takano T. (1996). Decrease of tissue angiotensin I-converting enzyme activity upon feeding sour milk in spontaneously hypertensive rats. Biosci. Biotech. Biochem..

[B50] Sipola M., Finckenberg P., Santisteban J., Korpela R., Vapaatalo H., Nurminen M.L. (2001). Long-term intake of milk peptides attenuates development of hypertension in spontaneously hypertensive rats. J. Physiol. Pharmacol..

[B51] Sipola M., Finckenberg P., Korpela R., Vapaatalo H, Nurminen M.L. (2002). Effect of long-term intake of milk products on blood pressure in hypertensive rats. J. Dairy Res..

[B52] Jauhiainen T., Collin M., Narva M., Cheng Z.J., Poussa T., Vapaatalo H, Korpela R. (2005). Effect of long-term intake of milk peptides and minerals on blood pressure and arterial function in spontaneously hypertensive rats. Milchwissenschaft.

[B53] Jäkälä P., Pere E, Lehtinen R., Turpeinen A., Korpela R., Vapaatalo H.  (2009). Cardiovascular activity of milk casein-derived tripeptides and plant sterols in spontaneously hypertensive rats. J. Physiol. Pharmacol..

[B54] Jäkälä P., Hakala A., Turpeinen A., Korpela R., Vapaatalo H. (2009). Casein-derived bioactive tripeptides Ile-Pro-Pro and Val-Pro-Pro attenuate the development of hypertension and improve endothelial function in salt-loaded Goto-Kakizaki rats. J. Functional Foods.

[B55] Jauhiainen T., Pilvi T., Cheng Z.J., Kautiainen H., Müller D.N., Vapaatalo H., Mervaala E. (2010). Milk products containing bioactive tripeptides have an antihypertensive effect in double transgenic rats (dTGR) harbouring human renin and human angiotensinogen genes. J. Nutr. Metab..

[B56] Masuda O., Nakamura Y., Takano T. (1996). Antihypertensive peptides are present in aorta after oral administration of sour milk containing these peptides to spontaneously hypertensive rats. J. Nutr..

[B57] Jäkälä P., Jauhiainen T., Korpela R., Vapaatalo H. (2009). Milk protein-derived bioactive tripeptides Ile-Pro-Pro and Val-Pro-Pro protect endothelial function *in vitro* in hypertensive rats. J. Functional Foods.

[B58] del Mar Contreras M., Carrón R., Montero M.J., Ramos M., Recio I. (2009). Novel casein-derived peptides with antihypertensive activity. Int. Dairy J..

[B59] Quirós A., Ramos M., Muguerza B., Delgado M.A., Miguel M., Aleixandre A., Recio I. (2007). Identification of novel antihypertensive peptides in milk fermented with *Enterococcus faecalis*. Int. Dairy J..

[B60] Antila P., Paakkari I., Järvinen A., Mattila M.J., Laukkanen M., Pihlanto-Leppälä A., Mäntsälä P., Hellman J. (1991). Opioid peptides derived from *in-vitro* proteolysis of bovine whey proteins. Int. Dairy J..

[B61] Nurminen M.L., Sipola M., Kaarto H., Pihlanto-Leppälä A., Piilola K., Korpela R., Tossavainen O., Korhonen H., Vapaatalo H. (2000). α-Lactorphin lowers blood pressure measured by radiotelemetry in normotensive and spontaneously hypertensive rats. Life Sci..

[B62] Sipola M., Finckenberg P., Vapaatalo H., Pihlanto-Leppälä A., Korhonen H., Korpela R., Nurminen M.-L. (2002). Alpha-lactorphin and beta-lactorphin improve arterial function in spontaneously hypertensive rats. Life Sci..

[B63] Murakami M., Tonouchi H., Takahashi R., Kitazawa H., Kawai Y., Negishi H., Saito T. (2004). Structural analysis of a new anti-hypertensive peptide (beta-lactosin B) isolated from a commercial whey product. J. Dairy Sci..

[B64] Abubakar A., Saito T., Kitazawa H., Kawai Y., Itoh T. (1998). Structural analysis of new antihypertensive peptides derived from cheese whey protein by proteinase K digestion. J. Dairy Sci..

[B65] Marshall K. (2004). Therapeutic applications of whey protein. Altern. Med. Rev..

[B66] Saito T. (2008). Antihypertensive peptides derived from bovine casein and whey proteins. Adv. Exp. Med. Biol..

[B67] Pripp A.H. (2008). Effect of peptides derived from food proteins on blood pressure: A meta-analysis of randomized controlled trials. Food Nutr. Res..

[B68] Xu J.Y., Qin L.Q., Wang P.Y., Li W., Chang C. (2008). Effect of milk tripeptides on blood pressure: A meta-analysis of randomized controlled trials. Nutrition.

[B69] Engberink M.F., Schouten E.G., Kok F.J., van Mierlo L.A.J., Brouwer I.A., Geleijnse J.M. (2008). Lactotripeptides show no effect on human blood pressure. Results from a double-blind randomized controlled trial. Hypertension.

[B70] van der Zander K., Bots M.L., Bak A.A.A., Koning M.M.G., de Leeuw P.W. (2008). Enzymatically hydrolyzed lactotripeptides do not lower blood pressure in mildly hypertensive subjects. Am. J. Clin. Nutr..

[B71] van der Zander K., Jakel M., Bianco V., Koning M.M. (2008). Fermented lactotripeptides-containing milk lowers daytime blood pressure in high normal-to-mild hypertensive subjects. J. Hum. Hypertens..

[B72] de Leeuw P.W., van der Zander K., Kroon A.A., Rennenberg R.M., Koning M.M. (2009). Dose-dependent lowering of blood pressure by dairy peptides in mildly hypertensive subjects. Blood Press..

[B73] Nakamura T., Mizutani J., Sasaki K., Yamamoto N., Takazawa K. (2009). Beneficial potential of casein hydrolysate containing Val-Pro-Pro and Ile-Pro-Pro on central blood pressure and hemodynamic index: A preliminary study. J. Med. Food.

[B74] Yoshizawa M., Maeda S., Miyaki A., Misono M., Choi Y., Shimojo N., Ajisaka R., Tanaka H. (2009). Additive beneficial effects of lactotripeptides and aerobic exercise on arterial compliance in postmenopausal women. Am. J. Physiol. Heart. Circ. Physiol..

[B75] Turpeinen A.M., Kumpu M., Rönnback M., Seppo L., Kautiainen H., Jauhiainen T., Vapaatalo H., Korpela R. (2009). Antihypertensive and cholesterol-lowering effects of a spread containing bioactive peptides IPP and VPP and plant sterols. J. Functional Foods.

[B76] van Mierlo L.A.J., Koning M.M.G., van der Zander K., Draijer R. (2009). Lactotripeptides do not lower blood pressure in untreated whites: Results from 2 controlled multicenter crossover studies. Am. J. Clin. Nutr..

[B77] Kajimoto O., Aihara K., Hirata H., Takahashi R., Nakamura Y. (2001). Hypotensive effects of tablets containing “lactotripeptides (VPP, IPP)”. J. Nutr. Food.

[B78] Seppo L., Kerojoki O., Suomalainen T., Korpela R. (2002). The effect of a *Lactobacillus helveticus* LBK-16H fermented milk on hypertension – a pilot study on humans. Milchwissenschaft.

[B79] Nakamura Y., Kajimoto O., Kaneko K., Aihara K., Mizutani J., Ikeda N., Nishimura A., Kajimoto Y. (2004). Effects of the liquid yogurts containing “lactotripeptide (VPP, IPP)” on high-normal blood pressure. J. Nutr. Food.

[B80] Aihara K., Kajimoto O., Hirata H., Takahashi R., Nakamura Y. (2005). Effect of powdered milk fermented with *Lactobacillus helveticus* on subjects with high-normal blood pressure or mild hypertension. J. Am. Coll. Nutr..

[B81] Mizuno S., Matsuura K., Gotou T., Nishimura S., Kajimoto O., Yabune M., Kajimoto Y., Yamamoto N. (2005). Antihypertensive effect of casein hydrolysate in a placebo-controlled study in subjects with high-normal blood pressure and mild hypertension. Br. J. Nutr..

[B82] Hirota T., Ohki K., Kawagishi R., Kajimoto Y., Mizuno S., Nakamura Y., Kitakaze M. (2007). Casein hydrolysate containing the antihypertensive tripeptides Val-Pro-Pro and Ile-Pro-Pro improves vascular endothelial function independent of blood pressure-lowering effects: Contribution of the inhibitory action of angiotensin-converting enzyme. Hypertens. Res..

[B83] Sano J., Ohki K., Higuchi T., Aihara K., Mizuno S., Kajimoto O., Nakagawa A., Kajimoto Y., Nakamura Y. (2005). Effect of casein hydrolysate, prepared with protease derived from *Aspergillus oryzae*, on subjects with high-normal blood pressure or mild hypertension. J. Med. Food.

[B84] Jauhiainen T., Vapaatalo H., Poussa T., Kyrönpalo S., Rasmussen M., Korpela R. (2005). Lactobacillus helveticus fermented milk lowers blood pressure in hypertensive subjects in 24-h ambulatory blood pressure measurement. Am. J. Hypertens..

[B85] Boelsma E., Kloek J. (2009). Lactotripeptides and antihypertensive effects: A critical review. Br. J. Nutr..

[B86] Pins J.J., Keenan J.M. (2006). Effects of whey peptides on cardiovascular disease risk factors. J. Clin. Hypertens. (Greenwich),.

[B87] Lee Y.M., Skurk T., Hennig M., Hauner H. (2007). Effect of a milk drink supplemented with whey peptides on blood pressure in patients with mild hypertension. Eur. J. Nutr..

[B88] Miguel M., Dávalos A., Manso M.A., de la Peña G., Lasunción M.A., López-Fandiño R. (2008). Transepithelial transport across Caco-2 cell monolayers of antihypertensive egg-derived peptides. PepT1-mediated flux of Tyr-Pro-Ile. Mol. Nutr. Food Res..

[B89] Foltz M., Cerstiaens A., van Meensel A., Mols R., van der Pijl P.C., Duchateau G.S.M.J.E. (2008). The angiotensin converting enzyme inhibitory tripeptides Ile-Pro-Pro and Val-Pro-Pro show increasing permeabilities with increasing physiological relevance of absorption models. Peptides.

[B90] Foltz M., Meynen E.E., Bianco V., van Platerink C., Koning T.M.M.G., Kloek J. (2007). Angiotensin converting enzyme inhibitory peptides from a lactotripeptide-enriched milk beverage are absorbed intact into the circulation. J. Nutr..

[B91] van der Pijl P.C., Kies A.K., Ten Have G.A.M., Duchateau G.S.M.J.E., Deutz N.E.P. (2008). Pharmacokinetics of proline-rich tripeptides in the pig. Peptides.

[B92] Vanhoof G., Goossens F., De Meester I., Hendriks D., Scharpé S. (1995). Proline motifs in peptides and their biological processing. FASEB J..

[B93] Jauhiainen T., Wuolle K., Vapaatalo H., Kerojoki O., Nurmela K., Lowrie C., Korpela R. (2007). Oral absorption, tissue distribution and excretion of a radiolabelled analog of a milk-derived antihypertensive peptide, Ile-Pro-Pro, in rats. Int. Dairy J..

[B94] Dolan E., Thijs L., Li Y., Atkins N., McCormack P., McClory S., O’Brien E., Staessen J.A., Stanton A.V. (2006). Ambulatory arterial stiffness index as a predictor of cardiovascular mortality in the Dublin Outcome Study. Hypertension.

[B95] Chirinos J.A., Zambrano J.P., Chakko S., Veerani A., Schob A., Willens H.J., Perez G., Mendez A.J. (2005). Aortic pressure augmentation predicts adverse cardiovascular events in patients with established coronary artery disease. Hypertension.

[B96] Jauhiainen T., Rönnback M., Vapaatalo H., Wuolle K., Kautiainen H., Korpela R. (2007). Lactobacillus helveticus fermented milk reduces arterial stiffness in hypertensive subjects. Int. Dairy J..

[B97] Jauhiainen T. (2007). Blood pressure lowering effects of *Lactobacillus helveticus* fermented milk containing bioactive peptides Ile-Pro-Pro and Val-Pro-Pro: Mechanistic, Kinetic and Clinical Studies.

[B98] Yamaguchi N., Kawaguchi K., Yamamoto N. (2009). Study of the mechanism of antihypertensive peptide VPP and IPP in spontaneously hypertensive rats by DNA microarray analysis. Eur. J. Pharmacol..

[B99] De Gennaro C.V., Rossoni G., Rigamonti A., Bonomo S., Manfredi B., Berti F., Muller E. (2002). Enalapril and quinapril improve endothelial vasodilator function and aortic eNOS gene expression in L-NAME-treated rats. Eur. J. Pharmacol..

[B100] Rummery N.M., Grayson T.H., Hill C.E. (2005). Angiotensin-converting enzyme inhibition restores endothelial but not medial connexin expression in hypertensive rats. J. Hypertens..

[B101] Fyhrquist F., Saijonmaa O. (2008). Renin-angiotensin system revisited. J. Intern. Med..

[B102] Wei L., Clauser E., Alhenc-Gelas F., Corvol P. (1992). The two homologous domains of human angiotensin I-converting enzyme interact differently with competitive inhibitors. J. Biol. Chem..

[B103] Natesh R., Schwager S.L.U., Sturrock E.D., Acharya K.R. (2003). Crystal structure of the human angiotensin-converting enzyme-lisinopril complex. Nature.

[B104] Meisel H. (2005). Biochemical properties of peptides encrypted in bovine milk proteins. Curr. Med. Chem..

[B105] Wuerzner G., Peyrard S., Blanchard A., Lalanne F., Azizi M. (2009). The lactotripeptides isoleucine-proline-proline and valine-proline-proline do not inhibit the N-terminal or C-terminal angiotensin converting enzyme active sites in humans. J. Hypertens..

[B106] Pripp A.H., Isaksson T., Stepaniak L., Sørhaug T. (2004). Quantitative structure-activity relationship modelling of ACE-inhibitory peptides derived from milk proteins. Eur. Food Res. Technol..

[B107] Wu J., Aluko R.E., Nakai S. (2006). Structural requirements of angiotensin I-converting enzyme inhibitory peptides: Quantitative structure-activity relationship study of di- and tripeptides. J. Agric. Food Chem..

[B108] Foltz M., van Buren L., Klaffke W., Duchateau G.S.M.J.E. (2009). Modeling of the relationship between dipeptide structure and dipeptide stability, permeability, and ACE-inhibitory activity. J. Food Sci..

[B109] FDA GRAS Substances (SCOGS) Database 2006.

[B110] (2009). Commission regulation (EC) No 983/2009. OJEU.

[B111] Piepho R.W. (2000). Overview of the angiotensin-converting-enzyme inhibitors. Am. J. Health Syst. Pharm..

[B112] Pryde P.G., Sedman A.B., Nugent C.E., Barr M. (1993). Angiotensin-converting enzyme inhibitor fetopathy. J. Am. Soc. Nephrol..

[B113] Quan A. (2006). Fetopathy associated with exposure to angiotensin converting enzyme inhibitors and angiotensin receptor antagonists. Early Hum. Dev..

[B114] Maeno M., Nakamura Y., Mennear J.H., Bernard B.K. (2005). Studies of the toxicological potential of tripeptides (L-valyl-L-prolyl-L-proline and L-isoleucyl-L-prolyl-L-proline): III. Single- and/or repeated-dose toxicity of tripeptides-containing *Lactobacillus helveticus*-fermented milk powder and casein hydrolysate in rats. Int. J. Toxicol..

[B115] Dent M.P., O’Hagan S., Braun W.H., Schaetti P., Marburger A., Vogel O. (2007). A 90-day subchronic toxicity study and reproductive toxicity studies on ACE-inhibiting lactotripeptide. Food Chem. Toxicol..

[B116] Ponstein-Simarro Doorten A.Y., vd Wiel J.A.G., Jonker D. (2009). Safety evaluation of an IPP tripeptide-containing milk protein hydrolysate. Food Chem. Toxicol..

[B117] (2007). Corrigendum to Regulation (EC) No 1924/2006 of the European Parliament and of the Council of 20 December 2006 on nutrition and health claims on foods.. OJEU.

